# Usefulness of Serum Leucine-rich Alpha 2 Glycoprotein in Crohn’s Disease: Is There Any Difference between Small Intestine and Colonic Lesions?

**DOI:** 10.1093/crocol/otad028

**Published:** 2023-05-23

**Authors:** Satohiro Matsumoto, Hirosato Mashima

**Affiliations:** From the Department of Gastroenterology, Jichi Medical University Saitama Medical Center, 1-847 Amanuma, Omiya, Saitama, Saitama 330-8503, Japan; From the Department of Gastroenterology, Jichi Medical University Saitama Medical Center, 1-847 Amanuma, Omiya, Saitama, Saitama 330-8503, Japan

**Keywords:** leucine-rich alpha-2 glycoprotein, cutoff value, delta value, mucosal healing, small intestine, Crohn’s disease

## Abstract

**Background:**

The usefulness of leucine-rich alpha 2 glycoprotein (LRG) to evaluate Crohn’s disease (CD) activity differs among various intestinal lesions. We aimed to evaluate the association between endoscopic disease activity based on the Simple Endoscopic Score for Crohn’s disease (SES-CD) and LRG level separately for small intestinal and colonic lesions.

**Methods:**

We examined the correlation between LRG level and SES-CD and performed receiver operating characteristic (ROC) analysis to determine the LRG cutoff value in 141 patients who underwent endoscopy (total 235 measurements). Furthermore, the LRG cutoff value was analyzed by comparing small intestinal and colonic lesions.

**Results:**

LRG levels were significantly higher in patients without mucosal healing than in those with mucosal healing (15.9 μg/mL vs 10.5 μg/mL, *P* < .0001). The LRG cutoff value for mucosal healing was 14.3 μg/mL (area under the ROC curve [AUC]: 0.80; sensitivity: 0.89; specificity: 0.63). The LRG cutoff value for patients with type L1 was 14.3 μg/mL (sensitivity: 0.91; specificity: 0.53), and that for patients with type L2 was 14.0 μg/mL (sensitivity: 0.95; specificity: 0.73). The diagnostic performance (AUC) of LRG and C-reactive protein (CRP) for mucosal healing was, respectively, 0.75 and 0.60 (*P* = 0.01) in patients with type L1 and 0.80 and 0.85 (*P* = 0.90) in patients with type L2.

**Conclusions:**

The optimal LRG cutoff value for evaluating mucosal healing in CD is 14.3 μg/mL. LRG is more useful than CRP for predicting mucosal healing in patients with type L1. The superiority of LRG to CRP differs between small intestinal and colonic lesions.

## Introduction

Crohn’s disease (CD) is a refractory inflammatory bowel disease (IBD) of unknown cause and with a progressive course because inflammation of the intestinal tract persists even without clinical symptoms and because CD causes intestinal complications such as stenosis and fistula in addition to ulcer. In the 2016 National Epidemiological Survey, the Health and Labour Sciences Research Group reported 220,000 patients registered with ulcerative colitis and 70,000 patients registered with CD. Although there are more patients with ulcerative colitis than with CD in Japan, the number of patients with CD is increasing every year.^1^

The biological agents used for CD treatment include anti-tumor necrosis factor (TNF)-α agents, anti-interleukin (IL) 12/23 antibodies, and anti-α4β7 integrin antibodies; moreover, anti-IL-23 antibodies were added in 2022. Therefore, therapeutic options for CD are greatly increasing. On the other hand, many patients require surgery because of intestinal complications such as stenosis and fistula. The cumulative surgery rates are reported to be 16.3% at 1 year, 33.3% at 5 years, and 46.6% at 10 years after the diagnosis of CD.^2^ Although the surgery rate has been decreasing because of the wide use of biological agents,^3^ the minimization of the need for surgery further requires regular control of disease activity and long-term remission maintenance therapy. The Selecting Therapeutic Targets in Inﬂammatory Bowel Disease (STRIDE-II) statement proposes clinical remission as an important medium-term therapeutic goal and mucosal healing as an important long-term therapeutic goal for patients with IBD.^4^ Since mucosal healing is closely associated with a shorter length of hospital stay and a lower surgery rate,^5,6^ therapeutic strategies aiming at mucosal healing are required.

To achieve mucosal healing, which is the target for drug efficacy evaluation, the disease activity needs to be maintained low by regular control of the disease activity based on clinical symptoms, such as diarrhea and abdominal pain, and on laboratory findings, such as C-reactive protein (CRP) level. CRP is an acute-phase protein that is synthesized in the liver in response to IL-6 stimulation. CRP is the most widely used surrogate marker for monitoring clinical disease activity in IBD. Although CRP is also an important serum marker for predicting mucosal healing,^7,8^ its usefulness for predicting mucosal healing in daily clinical practice is limited because the CRP cutoff value for predicting mucosal healing is low^9–11^ and because CRP measurements fluctuate in an extremely narrow range.

Leucine-rich alpha 2 glycoprotein (LRG), a new acute-phase protein, was identified based on the results of protein semi-quantitative analysis using serum samples collected from patients with rheumatoid arthritis before and after treatment with anti-TNF-α antibodies.^12^ Then, LRG was demonstrated to be useful for evaluating the disease activity of IBD. In June 2020, LRG was newly listed on the National Health Insurance Drug Price List in Japan. LRG has acute-phase protein properties that are similar to those of CRP and also properties that are different from those of CRP. For example, LRG synthesis is induced by stimulation with cytokines such as IL-1β, TNF-α, and IL-22 in addition to IL-6, and LRG is synthesized not only in the liver but also at the sites of inflammation.^13^ Although LRG has been reported to be useful for predicting mucosal healing in not only ulcerative colitis but also CD,^14–19^ there are some issues such as the lack of established cutoff values and the uncertainty about differences in the usefulness of LRG among disease types and between the small intestinal and colonic lesions. In this study, we analyzed LRG levels and endoscopic disease activity in patients with CD to determine appropriate LRG cutoff values for mucosal healing and to evaluate the differences in the usefulness of LRG between small intestinal and colonic lesions.

## Materials and Methods

### Subjects

This was a retrospective single-center study at Saitama Medical Center. Data of 154 Japanese patients with CD who received outpatient treatment at Saitama Medical Center between July 2020 and July 2022 and whose serum LRG levels were measured at least once were extracted from medical records. Patients who had an evident infection, extraintestinal complications other than mild ones, or anal fistula lesions at the time of blood sampling for LRG measurement and patients who received a coronavirus disease 2019 vaccine in the previous 1 week were excluded. Nanopia LRG^®^ (Sekisui Medical, Tokyo, Japan) was used for LRG measurements. A total of 892 LRG measurements were performed in the 154 patients. First, the association between LRG level and clinical disease activity was analyzed. During the study period, endoscopy was performed in all 154 patients (total 291 sessions). From these 154 patients, we selected 141 patients (total 235 sessions) who underwent ileocolonoscopy or transanal enteroscopy within 2 months before and after LRG measurement and had no changes in disease condition or treatment during the same period. In these patients, we analyzed the association between LRG level and endoscopic disease activity to determine the optimal cutoff value for predicting mucosal healing. While the normal range of CRP was defined as less than 2 mg/L, the association between LRG and endoscopic disease activity was analyzed in CRP-negative patients. Next, to examine the differences in the usefulness of LRG between small intestinal and colonic lesions, the association between the optimal LRG cutoff value for predicting mucosal healing and endoscopic disease activity was analyzed separately for each type based on the disease location.

This study was approved by The Etiological Study Ethical Review Board of Jichi Medical University Saitama Medical Center. Because we produced anonymized data and used them, the need for informed consent was waived.

### Assessment of Clinical and Endoscopic Disease Activity

Clinical symptoms were scored using the Crohn’s Disease Activity Index (CDAI), and a CDAI score of less than 150 was considered to indicate clinical remission. Relapse was defined as an increase in CDAI from less than 150 to more than 150.^20^ The Simple Endoscopic Score for Crohn’s Disease (SES-CD) is based on the evaluation of four components (ie, ulcer size, proportion of ulcer area, proportion of lesion area, and stenosis), each of which is scored on a 0- to 3-point scale separately in five segments (ie, ileum, right colon, transverse colon, left colon, and rectum). The SES-CD is calculated as the sum of all component scores (0–56 points).^21^ In this study, stenosis without ulcer was scored as 0 points because it is a morphological change of the intestine and is not caused by current inflammation.^22^ CD severity was defined as inactive when SES‑CD was 0–2; mild, when it was 3–6; moderate, when it was 7–15; and severe, when it was ≥16.^23^ Mucosal healing was defined as an SES-CD of 0. All endoscopic sessions were performed by experienced endoscopists, and endoscopic results were scored by investigators who were blinded to hematologic test results. In some patients with small intestinal-to-colonic type CD, small intestinal lesions were evaluated by capsule endoscopy. We excluded the results of capsule endoscopy from the analysis.

### Statistical Analysis

Categorical variables are expressed as numbers of subjects (percentages), while continuous variables are expressed as medians (ranges) and compared using the Mann–Whitney *U* test and the Kruskal–Wallis test with post hoc Bonferroni analysis. The Spearman’s rank correlation coefficient was used to measure the strength and direction of the correlation between two variables. The receiver operating characteristic (ROC), is a plot of true positive fraction vs. false positive fraction, and area under the ROC curve (AUC) was calculated to determine the optimal cutoff value. All statistical analyses were performed using EZR version 1.54 (Jichi Medical University Saitama Medical Center, Shimotsuke, Japan).^24^ Differences with *P* values < 0.05 were regarded as statistically significant.

## Results

### LRG Concentration in Relation to Clinical Activity

**Figure 1.**
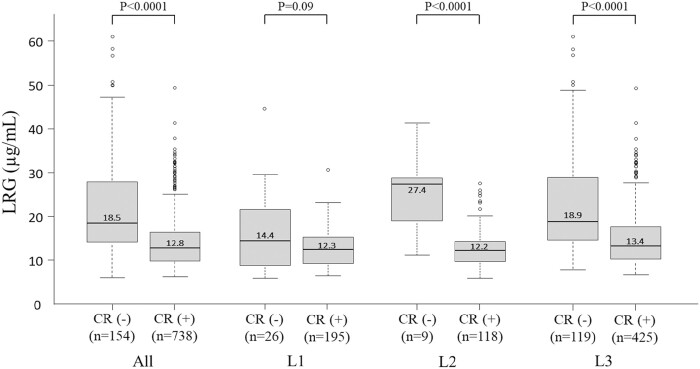
Serum LRG levels in patients with CD categorized according to disease activity and disease location (*n* = 892, cumulative total number of LRG measurements). LRG, leucine-rich alpha-2 glycoprotein; CD, Crohn’s disease.

Table 1 shows patient characteristics. The median age of patients was 42 years (range, 16–85 years). According to disease type, the small intestinal type (L1) accounted for 31.2% of patients, the colonic type (L2) for 21.4%, and the small intestinal-to-colonic type (L3) for 47.4%. LRG levels showed a strong positive correlation with CRP level (correlation coefficient: 0.76; 95% confidence interval [CI]: 0.73–0.79; *P* < .0001) and moderate negative correlation with albumin (Alb) level (correlation coefficient: −0.69; 95% CI: −0.73 to −0.66; *P* < .0001). Weak positive correlation was observed between serum LRG level and CDAI (correlation coefficient: 0.47; 95% CI: 0.41–0.52; *P* < .01). When LRG levels (total 892 measurements) were compared between patients with CD in clinical remission and patients with active CD, LRG levels were higher in patients with active CD than in patients in clinical remission (18.5 μg/mL vs 12.1 μg/mL; *P* < .0001). According to disease type, serum LRG levels were higher in those with active CD than in those in clinical remission in patients with type L2 or L3, but no significant difference was observed in patients with type L1 (Figure 1).

**Table 1.** Baseline characteristics.

	All patients (*n* = 154)	Subjects (*n* = 141)
Male, number	111 (72.1%)	103 (73.0%)
Age at onset, y	26 (0−82)	26 (0−82)
Age at entry, y	42 (16−85)	42 (16−85)
Duration of ulcerative colitis, y	8.8 (0−41.1)	8.7 (0−41.1)
Disease location		
Ileum (L1)	48 (31.2%)	43 (30.5%)
Colon (L2)	33 (21.4%)	31 (22.0%)
Ileum and colon (L3)	73 (47.4%)	67 (47.5%)
Upper GI tract inflammation	33 (21.4%)	29 (20.6%)
Disease phenotype		
Non-structuring, non-penetrating (B1)	70 (45.5%)	62 (44.0%)
Stricturing (B2)	57 (37.0%)	54 (38.3%)
Penetrating (B3)	27 (17.5%)	25 (17.7%)
Extraintestinal manifestations	29 (18.8%)	26 (18.4%)
Anal fistula	25 (16.2%)	23 (16.3%)
Prior ileocolonic resection	42 (27.3%)	37 (26.2%)
Current smoking	24 (15.6%)	22 (15.6%)
Prior treatment		
Corticosteroid	98 (63.6%)	90 (63.8%)
Cytapheresis	4 (2.6%)	4 (2.8%)
Immunomodulators	51 (33.1%)	44 (31.2%)
Biologics	30 (19.5%)	26 (18.4%)
Concomitant treatment		
Mesalazine	136 (88.3%)	124 (87.9%)
Immunomodulators	41 (26.6%)	37 (26.2%)
Biologics	111 (72.1%)	103 (73.0%)
Infliximab	38 (24.7%)	35 (24.8%)
Adalimumab	40 (26.0%)	38 (27.0%)
Ustekinumab	32 (20.8%)	29 (20.6%)
Vedolizumab	1 (0.6%)	1 (0.7%)
CDAI	59.7 (0−465.3)	64.0 (0−416.0)
LRG (μg/mL)	13.5 (5.2−61.2)	12.9 (5.3−58.3)
C-reactive protein (mg/L)	1.3 (0.1−108.9)	1.0 (0.1−73.0)
Albumin (g/dL)	4.2 (2.2−5.3)	4.2 (2.7−5.1)
SES-CD	NA	3 (0−23)

Data are presented as median (range) or number (%). GI, gastrointestinal; CDAI, Crohn’s disease activity index; LRG, leucine-rich alpha-2 glycoprotein; SES-CD, simple endoscopic score for Crohnʼs disease

### LRG Concentration in Relation to Endoscopic Activity

**Figure 2.**
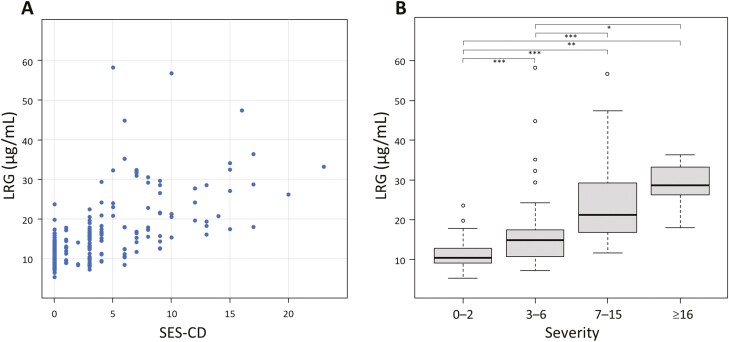
Serum LRG levels plotted according to endoscopic activity in patients with CD (A). Serum LRG levels according to the severity of CD (B). *P < .05; **P < .01; ***P < .001. LRG, leucine-rich alpha-2 glycoprotein; CD, Crohn’s disease; SES-CD, applied Simple Endoscopic Score for Crohn’s disease.

**Figure 3.**
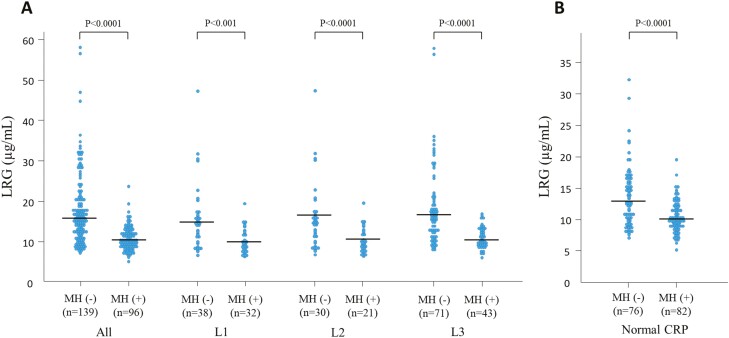
Serum LRG levels between the MH group and non-MH group in patients with CD categorized according to disease location (A) (*n* = 235, cumulative total number of patients undergoing endoscopy). Serum LRG levels between the MH group and non-MH group in patients with CD with normal CRP levels (< 2 mg/L) (B). LRG, leucine-rich alpha-2 glycoprotein; MH; mucosal healing; CD, Crohn’s disease; CRP, C-reactive protein.

We evaluated whether LRG was associated with endoscopic disease activity in patients with CD (141 patients with total of 235 measurements). The interval between endoscopy and LRG measurement was 35.4 ± 18.5 days (range, 0–60 days). LRG levels steadily increased as SES-CD increased. Inflammation of the intestinal mucosa was significantly correlated with increased LRG levels (correlation coefficient: 0.63; 95% CI: 0.55–0.70; *P* < .0001) (Figure 2A). In all patients with an LRG level of less than 7.2 μg/mL, SES-CD was 0 so that endoscopically active disease was not observed. As shown in Figure 2B, LRG serum levels significantly increased as the endoscopic disease activity increased (*P* < .0001). LRG serum levels were significantly lower in patients with an SES-CD score of 0–2 compared with those in patients with higher scores for any segment. In all disease types, a significant positive correlation was observed between serum LRG levels and SES-CD: L1 (correlation coefficient: 0.77; 95% CI: 0.65–0.85; *P* < .0001), L2 (correlation coefficient: 0.63; 95% CI: 0.42–0.77; *P* < .0001), and L3 (correlation coefficient: 0.59; 95% CI: 0.45–0.70; *P* < .0001). LRG levels were significantly higher in patients without mucosal healing than in those with mucosal healing (15.9 μg/mL vs 10.5 μg/mL, *P* < .0001). Similar results were obtained for all disease types (Figure 3A). In addition, even among patients with CD and normal CRP levels (CRP < 2 mg/L), LRG levels were significantly higher in those without mucosal healing than in those with mucosal healing (Figure 3B).

### Cutoff Value of LRG and CRP for Evaluation of Mucosal Healing in Patients with CD

**Figure 4.**
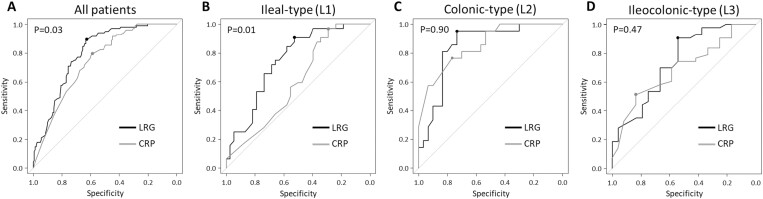
The receiver operating characteristic curve of LRG and CRP for predicting mucosal healing. All patients (A), ileal-type (B), colonic-type (C), and ileocolonic-type (D). LRG, leucine-rich alpha-2 glycoprotein; CRP, C-reactive protein.

Figure 4 shows ROC curves showing the abilities of LRG and CRP to detect mucosal healing. For detection of mucosal healing (SES-CD: 0), AUC of LRG was 0.80 (95% CI: 0.74–0.85) with a cutoff value of 14.3 μg/mL, and AUC of CRP was 0.74 (95% CI: 0.68–0.80) with a cutoff value of 1.3 mg/L (*P* = 0.03) (Figure 4A). The diagnostic accuracy of LRG for mucosal healing was calculated as sensitivity of 0.89, specificity of 0.63, positive predictive value of 0.63, and negative predictive value of 0.89. When the CRP cutoff value was set at 2 mg/L, which is considered to be within the normal range, the diagnostic accuracy was calculated as sensitivity of 0.85, specificity of 0.45, positive predictive value of 0.52, and negative predictive value of 0.82.

### Differences among the Types of Disease Locations

To examine whether LRG levels differed between inflammation of the small intestine and colon, patients were divided into three types based on the disease location (type L1, type L2, and type L3) and analyzed. The SES-CD in patients with type L2 was the highest (L1, 1; L2, 3; L3, 0; *P* < .001). CDAI, LRG, and CRP showed no significant differences among the three groups (Table 2). Based on the ROC curves showing the abilities of LRG and CRP to detect mucosal healing, in patients with type L1, the AUC of the LRG was 0.75 (95% CI: 0.63–0.86) with a cutoff value of 14.3 μg/mL (sensitivity: 0.91; specificity: 0.53), and the AUC of the CRP was 0.60 (95% CI: 0.46–0.73) with a cutoff value of 2.0 mg/L (sensitivity: 0.97; specificity: 0.29). LRG was superior to CRP (*P* = 0.01) (Figure 4B). In patients with type L2, however, the AUC of the LRG was 0.80 (95% CI: 0.74–0.85) with a cutoff value of 14.0 μg/mL (sensitivity: 0.95; specificity: 0.73), and the AUC of the CRP was 0.85 (95% CI: 0.75–0.96) with a cutoff value of 0.8 mg/L (sensitivity: 0.76; specificity: 0.77). The superiority of LRG was not observed (*P* = 0.90) (Figure 4C). In patients with type L3, the AUC of the LRG was 0.74 (95% CI: 0.61–0.87) with a cutoff value of 14.3 μg/mL (sensitivity: 0.91; specificity: 0.54), and the AUC of the CRP was 0.69 (95% CI: 0.56–0.82) with a cutoff value of 0.5 mg/L (sensitivity: 0.51; specificity: 0.83). The superiority of LRG was not observed (*P* = 0.47) (Figure 4D).

**Table 2.** Disease activity and serum biomarker among the types of disease location.

	Type L1 (*n* = 70)	Type L2 (*n* = 51)	Type L3 (*n* = 114)	*P* value
CDAI	49.3 (0−396.3)	62.9 (0−250.2)	56.3 (0−416.0)	0.74
LRG (μg/mL)	12.2 (6.9−47.4)	12.7 (5.3−44.9)	11.9 (6.4−58.3)	0.15
C-reactive protein (mg/L)	0.8 (0.1−37.3)	1.1 (0.1−20.8)	0.7 (0.1−54.0)	0.27
Albumin (g/dL)	4.4 (2.9−5.1)	4.2 (2.7−5.1)	4.2 (2.9−5.1)	0.39
SES-CD	1 (0−8)	3 (0−17)	0 (0−13)	<0.001

Data are presented as median (range). CDAI, Crohn’s disease activity index; LRG, leucine-rich alpha-2 glycoprotein; SES-CD, simple endoscopic score for Crohn’s disease

### Monitoring Using LRG during the Clinical Course of Patients with CD

**Figure 5.**
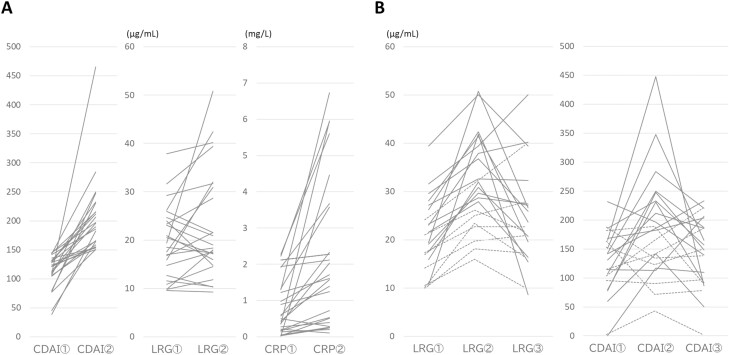
Transitional change of clinical and biomarker. Transitional change of CDAI, LRG, and CRP at the two consecutive points in patients with relapsed CD (A). Transitional change of LRG and CDAI at the three consecutive points in patients with LRG elevation of 5 μg/mL or more than the previous value (B). Cases with (solid line) or without (dotted line) intensified treatment at the time of ②. CD, Crohn’s disease; LRG, leucine-rich alpha-2 glycoprotein; CRP, C-reactive protein; CDAI, Crohn’s disease activity index.

During the observation period, 24 relapsing episodes occurred in 19 patients (12.3%). LRG levels were elevated in 58.3% of these episodes (14/24), and the mean and median delta values of LRG were 3.5 and 2.2 μg/mL, respectively. CRP levels were elevated in 58.3% (14/24), and the mean and median delta values of CRP were 6.3 and 0.3 mg/L, respectively (Figure 5A). Next, patients whose LRG levels increased by 5 μg/mL or more from the previous level were examined. There were 17 such patients (11.0%) with 22 relapsing episodes. Exacerbation of symptoms and an increase in CDAI scores were observed in 63.6% of the episodes (14/22). In 15 of the 22 episodes, treatment was intensified. In the remaining seven episodes, patients were followed up without intensification of treatment. Subsequently, CD was exacerbated in two of these patients. In these two patients, the delta values of LRG before and after relapse were 7.6 and 8.2 μg/mL, respectively (Figure 5B).

## Discussion

This study found an optimal LRG cutoff value for evaluating mucosal healing in CD of 14.3 μg/mL. However, this indicator was more useful than CRP for predicting mucosal healing in the ileum dominant group, suggesting that its utility differs in various intestinal lesions.

In CD, intestinal complications such as stenosis and fistula greatly affect the quality of life of patients so that prevention of stenosis and fistula is an important element of therapeutic strategies for CD. Strategies that prevent these conditions can eventually lead to avoidance of surgery. A meta-analysis reported that mucosal healing can be beneficial for reducing the risk of surgery.^6^ Because the concept of setting mucosal healing as the therapeutic goal has been generally accepted, the methods to monitor for mucosal healing have attracted attention. Although endoscopy is an essential method to evaluate mucosal healing, it is not only invasive and difficult to perform frequently, but it may also exacerbate symptoms of patients with CD.^25^ Thus, reliable noninvasive surrogate markers are needed as an alternative to endoscopic evaluation.

In the past, CRP has mainly been used for monitoring patients with CD on maintenance therapy. CRP has been reported to be useful for predicting mucosal healing in CD.^7,8^ In patients with CD treated with biological agents, the CRP cutoff value for predicting mucosal healing at week 52 was 4.2 mg/L (sensitivity: 0.95; specificity: 0.64).^19^ We previously reported that CRP level at the introduction of anti-TNFα agents is a predictor of reduced therapeutic effects in patients with CD,^26^ and that CRP/Alb ratio at 3 months after the introduction of anti-TNFα agents is a predictor of secondary failure.^27^ A previous report on patients with CD who started treatment with anti-TNFα agents showed that treat-to-target strategies based on CRP or Alb were useful for preventing hospitalization, surgery, and discontinuation of biological therapy for CD.^28^ Thus, a combination of CRP and Alb, which are simple biomarkers, can improve the ability to predict mucosal healing. However, CRP is difficult to use in daily clinical practice because of its low cutoff value.^9–11^ Moreover, in a study in which magnetic resonance enterography and balloon endoscopy were performed in patients with CD in clinical remission whose CRP level was less than 3 mg/L, active lesions were detected in the small intestine in 45% of all patients.^29^ This finding indicates that CRP is unlikely to reflect disease activity of small intestinal lesions. Among surrogate markers to evaluate mucosal inflammation, fecal calprotectin was found to significantly correlate with endoscopic disease activity,^30^ and the STRIDE-II guidelines recommend a fecal calprotectin level of 100–250 μg/g as a medium-term therapeutic goal.^4^ However, fecal markers have several problems, including diurnal variation in measurements, insufficient collection of stool samples, and the lack of established cutoff values.^31^

There is no defined cutoff value of fecal calprotectin, and most studies use cutoff values based on ROC curves. On the other hand, the reference value of LRG is considered to be 16.0 μg/mL; however, reports on cutoff values for predicting mucosal healing in CD are extremely limited. Yasutomi et al. reported that the LRG cutoff value for predicting mucosal healing (SES-CD: 0) in patients with CD was 13.0 μg/mL (sensitivity: 0.84; specificity: 0.73).^15^ Kawamura et al., who performed peroral and transanal balloon endoscopy in patients with CD, reported that the LRG cutoff value for predicting mucosal healing (SES-CD: 0–2) was 8.9 μg/mL (sensitivity: 0.93; specificity: 0.83).^17^ In patients with CD treated with biological agents, the LRG cutoff value for predicting mucosal healing at week 52 was 13.6 μg/mL (sensitivity: 0.85; specificity: 0.89).^19^ In the current study, an LRG level of 14.3 μg/mL was identified as a cutoff value for mucosal healing (SES-CD: 0), but the diagnostic accuracy of LRG was found to differ between small intestinal and colonic lesions.

Small intestinal lesions are detected in approximately 70% of patients with CD.^32^ A Japanese report also indicated that small intestinal lesions are detected in 82% of patients with CD.^33^ Active small intestinal lesions are independently associated with poor prognosis in patients with CD,^29^ and extensive small intestinal lesions are demonstrated to be a factor for poor prognosis of CD.^34^ Thus, the presence of small intestinal lesions is an important element of therapeutic strategies for CD. However, because CD is sometimes associated with stenosis of particularly the small intestine, endoscopic evaluation is often difficult. Thus, biomarkers that can predict mucosal healing in the small intestine may be valuable for therapeutic strategies for small intestinal lesions. In a study that focused on small intestinal lesions of CD and evaluated balloon endoscopic findings with modified SES-CD, the LRG cutoff value for predicting the presence of an ulcer measuring 5 mm or more was 13.4 μg/mL (sensitivity: 0.79; specificity: 0.82).^18^ In another study that evaluated small intestinal lesions of CD with capsule endoscopy, the LRG cutoff value for predicting the presence of an ulcer measuring 5 mm or more was 14.0 μg/mL (sensitivity: 0.64; specificity: 0.83).^33^ The current study showed that, in terms of accuracy in predicting mucosal healing, LRG was superior to CRP in patients with type L1, but comparable to CRP in patients with type L2. Thus, for the ileal type, monitoring of both LRG and CRP is necessary, instead of monitoring of CRP alone.

For monitoring of mucosal status, we consider that the delta value of LRG is also important in addition to the LRG cutoff value. In a study of patients with IBD who started treatment with adalimumab, Shinzaki et al. reported that LRG levels were significantly lower at all measurement points during a 52-week period in patients with endoscopic remission than in patients without endoscopic remission, demonstrating the suitability of LRG for long-term monitoring.^14^ The current study showed that, although LRG levels were not elevated in approximately 40% of patients who experienced relapse, clinical symptoms were exacerbated in 60% of patients whose LRG levels were elevated from the previous level by 5 μg/mL. On the other hand, among the patients with an LRG level elevated by 5 μg/mL, there were some patients who were only followed up without intensification of treatment and had no problems with clinical symptoms. Thus, future issues include how delta values of LRG should be evaluated and interpreted and whether treatment of asymptomatic patients should be intensified based on elevated LRG levels alone. These issues are difficult to solve using LRG level alone, and comprehensive judgment based on measurements of CRP, Alb, etc. may be necessary.

The current study has limitations. It is a single-center retrospective study. In most study patients, LRG measurement and endoscopy were performed on different days. Endoscopic observation was performed with ileocolonoscopy up to the end of the ileum in many patients. In patients who had lesions confirmed on the oral side from the end of the ileum at or after the time of diagnosis, balloon endoscopy or capsule endoscopy in combination with ileocolonoscopy was performed to enable observation of the small intestine as soon as possible. CD is a transmural disease, and endoscopy does not fully capture the disease’s extent or burden. It has been reported that transmural healing evaluated by magnetic-resonance enterography^35^ or ultrasonography^36^ is associated with better outcomes than endoscopic mucosal healing in CD. Therefore, some patients with SES-CD 0 may have transmural inflammation. Since the current study was based on an examination of as many as approximately 900 LRG measurements, we consider it to be a valuable study.

## Conclusion

LRG levels and endoscopic scores of CD are positively correlated, and the optimal LRG cutoff value for evaluating mucosal healing is 14.3 μg/mL. Although LRG is not superior to CRP in predicting mucosal healing in patients with type L2, it is more useful for predicting mucosal healing than CRP in patients with type L1. However, because of the low specificity and high false positive rate of LRG, the delta value may also be useful for monitoring of LRG levels in addition to the cutoff value.

## Data Availability

Data are not publicly available.
